# Experiences from a pilot study on how to conduct a qualitative multi-country research project regarding use of antibiotics in Southeast Europe

**DOI:** 10.1186/s40545-016-0069-3

**Published:** 2016-05-23

**Authors:** Susanne Kaae, Sofia Kälvemark Sporrong, Janine Morgall Traulsen, Helle Wallach Kildemoes, Lotte Stig Nørgaard, Arianit Jakupi, Denis Raka, Emre Umut Gürpinar, Ali Alkan, Iris Hoxha, Admir Malaj, Lourdes Arevalo Cantarero

**Affiliations:** Section for Social and Clinical Pharmacy, Department of Pharmacy, Faculty of Health and Medical Sciences, University of Copenhagen, Universitetsparken 2, 2100 København Ø, Denmark; A2 Pharmaceutical Consulting, Mother Teresa boulevard, B1, No:19, Prishtinë, Republika e Kosovës; Medical Faculty, Pharmacy Department, University of Prishtina, Bulevardi Dëshmorët e Kombit, p.n., 10000 Prishtinë, Republika e Kosovës; Turkish Medicines and Medical Devices Agency, Söğütözü Mahallesi 2176. Sokak, No:5 PK:06520, Çankaya/Ankara, Turkey; Faculty of Pharmacy, University of Medicine Tirana, Albania, Fakulteti Farmacise, Rr. Dibres 371, 1000 Tirana, Albania

**Keywords:** Qualitative methodology, Antibiotic use culture, Multi-country project

## Abstract

**Background:**

In 2014, a qualitative multi-country research project was launched to study the reasons behind the high use of antibiotics in regions of Southeast Europe by using previously untrained national interviewers (who were engaged in other antibiotic microbial resistance-related investigations) to conduct qualitative interviews with local patients, physicians and pharmacists. Little knowledge exists about how to implement qualitative multi-country research collaborations involving previously untrained local data collectors. The aim of this paper was therefore to contribute to the knowledge regarding how to conduct these types of research projects by evaluating a pilot study of the project.

**Methods:**

Local data collectors conducted the study according to a developed protocol and evaluated the study with the responsible researcher-team from University of Copenhagen. The pilot study focused on ‘local ownership’, ‘research quality’ and ‘feasibility’ with regard to successful implementation and evaluation. The evaluation was achieved by interpreting ‘Skype’ and ‘face to face’ meetings and email correspondence by applying ‘critical common sense’.

**Results:**

Local data collectors achieved a sense of joint ownership. Overall, the protocol worked well. Several minor challenges pertaining to research quality and feasibility were identified, in particular obtaining narratives when conducting interviews and recruiting patients for the study. Furthermore, local data collectors found it difficult to allocate sufficient time to the project. Solutions were discussed and added to the protocol.

**Conclusions:**

Despite the challenges, it was possible to achieve an acceptable scientific level of research when conducting qualitative multi-country research collaboration under the given circumstances. Specific recommendations to achieve this are provided by the authors.

## Background

For decades, a considerable number of research collaborations have been launched between western and non-western partners with the aim of improving health in emerging economies. Several critical factors have been identified in this regard, including (too little) attention to local priorities, feasibility, capacity building, pilot studies, stakeholder support, refinement of research protocols and information systems, and ethical issues [1–3].

Research on antimicrobial resistance is one contemporary health area in which partnerships between different countries have been established. For example, the World Health Organization (WHO) Regional Office for Europe has undertaken numerous initiatives in different locations, including in the region of Southeastern Europe. One initiative includes the continuous surveillance of antibiotic (AB) drug consumption. Systematic surveillance measurements and publications of antimicrobial consumption within the European setting began with the ESAC initiative with EU countries [4], but data on AB consumption are now also being collected through the management of the Health Technologies and Pharmaceuticals group (HTP), the WHO Regional office for Europe in non-EU Southeast European countries. Through this effort, it has been documented that a high level of inappropriate AB use is present in this region [5].

To determine the reasons for inappropriate AB use and trends in consumption in this area, it has been suggested to supplement consumption data with additional data on prescriptions and to collect detailed information about national programmes and campaigns on the prudent use of antimicrobials [6]. Another useful approach to understanding the reasons for the inappropriate use of AB is to apply qualitative research. Hence, in 2014, an additional qualitative research project in the geographical area of Southeast Europe was launched with the aim of investigating the AB use culture, i.e., the knowledge, attitudes and actual behaviours of patients and health care practitioners, to inform individual countries on how to target future interventions. Countries already engaged in the WHO AB consumption data collection group in the non-EU Southeast European region were invited to take part in the qualitative project with the role of becoming the primary data collectors i.e., local project facilitators. Originally, 17 countries participated in the AB consumption data collecting group [5], and the group has now been expanded to include 19 countries in total.

There are many advantages to having local countries conduct qualitative research. First, there are no language barriers. This enables a dialogue with all relevant stakeholders in the country without the use of interpreters, as the use of interpreters has been shown to reduce validity [7]. Another advantage is that locally based research is more sensitive to capturing local practices and ideas. However, in qualitative studies, the researcher becomes the data collection instrument. In general, the results therefore depend more on the skills of the researcher when compared with quantitative instruments such as surveys, in which questions and categories of answers are pre-determined and standardized.

The qualitative project was managed by researchers from the Section of Social and Clinical Pharmacy at the University of Copenhagen (SSC). The SSC has decades of experience in conducting research projects employing qualitative methods within the field of medicine use [8]. Knowledge exists on how to develop research collaborations between partners, such as academic researchers and practitioners, in different countries [9, 10], including for qualitative projects [11]. However, we found little information regarding how to implement qualitative research collaborations involving up to 19 countries using local data collectors, most of whom were expected to be previously untrained in qualitative methods.

The aim of this paper is therefore to contribute to the knowledge on how to conduct qualitative multi-country research projects under the aforementioned settings. The information was obtained through a pilot study and therefore illustrates our experiences conducting the qualitative AB project in Albania, Kosovo and Turkey.

## Methods

In our attempt to ensure successful implementation of the qualitative AB multi-country project, we decided to focus on three particular factors [10]:

- Local ownership (acceptability)
- Research quality (implementation)
- Feasibility (practicalities)

Local ownership was a priority to ensure that local project facilitators considered the project relevant and that they could achieve valuable knowledge by participating in the project. Because the goal of the project was to enable countries to make informed interventions, it was also essential that the research results were of an acceptable quality. Finally, feasibility was important. Most facilitators had no previous experience conducting qualitative research and were often busy carrying out other jobs simultaneously. As a result, it was necessary to develop and evaluate these elements through a pilot study.

### Measures to ensure local ownership, research quality and feasibility

To generate local commitment, the first step of the project took place in 2014 in Slovenia when the project was presented to a group of countries in the Balkan area of Southeastern Europe. Seven countries participated, and the attendees were primarily health care professionals (typically pharmacists and pharmacologists) working at national medicine agencies and universities. Three countries volunteered to run the pilots: Albania, Kosovo and Turkey.

The SSC decided that semi-structured interviews should be the applied method for investigating AB knowledge, behaviour and attitudes of patients and health care professionals. The SSC made several other initial decisions on how to conduct the study, taking into consideration the aspects of local ownership, research quality and feasibility (please see Table 1) [5, 12–14]. The decisions were accounted for in a protocol to align the research conducted by the local facilitators in different countries.

**Table 1:** Initial decisions and rationale to ensure ownership, research quality and feasibility (before conducting the pilot evaluation)

Decision	Rationale with respect to: local ownership/feasibility/research quality
Data collection and transcriptions should be completed by national project facilitators	*Local ownership* Local participants carry out and transcribe the conducted interviews to create local ownership and specific insight into how future interventions could be targeted.
General practitioners (GP) and community pharmacists who prescribe or sell antibiotics should be interviewed	*Research quality* Interviews with GPs were supplemented with interviews with community pharmacists due to their role in dispensing/informing patients about antibiotics in general and the documented practice of providing antibiotics without a prescription in community pharmacies in some countries in Southeast Europe (Hoxha et al.).
Patients interviewed should be adults who have suffered from an infection within the previous 3 months that they treated with antibiotics (with or without a prescription)	*Research quality* Adults were chosen to ensure a minimum comparability of data (antibiotic use by children is believed to contain other aspects, especially regarding attitudes – Wun et al.). A 3 month limit was selected to reduce memory bias. Both patients with and without prescriptions should be included, as the high antibiotic consumption could be caused by both groups (Mitsi et al.).
Infections should consist of only upper respiratory tract infections	*Research quality* The area of infection was specified to ensure comparability of data. Furthermore, upper respiratory tract infections are common diseases, hence constituting an ideal case to study the typical reasons behind high antibiotic consumption with regard to knowledge, attitudes and behaviour.
Specific antibiotics to be investigated were: amoxicillin-clavulanic acid, azithromycin, ciprofloxacin and ceftriaxone	*Research quality* Specific antibiotics were selected to ensure the comparability of data. The four antibiotics selected have all been shown to be used irrationally in the region of Southeastern Europe (Versporten et al.), with a risk to public health, as most are broad spectrum. A few deviations to this could be tolerated if the ABs were not used in a specific country.
Three interviews should be conducted within the four groups of interviewees: patients with a prescription, patients without a prescription, GPs and community pharmacists; hence, 12 interviews total per country. The patient interviews should contain a detailed description of the last time the interviewee was prescribed/bought an antibiotic. Interviews with health care professionals should address detailed descriptions of 3 specific episodes during the last week in which they handled antibiotics. All specific incidents should further be explored in relation to the way the relevant parties usually prescribe/purchase or prescribe/sell antibiotics	*Feasibility and research quality* The number of interviews was designed to maintain a decent workload balance for the participants, yet it was expected that it would be possible through data triangulation of the 12 interviews to detect condensed patterns of antibiotic knowledge, attitudes and behaviour. Antibiotic knowledge, behaviour and attitudes can best be derived through detailed narratives, which are more easily obtained when they address specific recent incidents. To ensure that this recent incident represented typical behaviour, its resemblance to former similar situations was also explored. As the number of AB episodes described in the interviews that would be used in the data analysis was therefore at least 24 (6 from patients and 18 from health care professionals), it was estimated that data saturation could then be achieved regarding aspects of AB use.
Data analysis should be conducted in collaboration between the Section for Social and Clinical Pharmacy and local facilitators	*Research quality* Combining the analytical competencies of the researchers from Copenhagen with detailed knowledge of the local culture of local facilitators was assessed as being the most ideal way of achieving high quality results.

To adequately assess the culture of patients and health care professionals as it related to AB use, the protocol specified the themes within AB use that were to be explored in the interviews. The protocol further specified the research questions pertaining to each of these themes and contained the operationalized forms of these research questions, i.e., the interview guides (please see Table 2 as well as Appendix 1 for one example of an interview guide). The themes were based on previous literature [15] and discussions with experts in the field of rational AB use (please see acknowledgements).

**Table 2:** Relation between research themes, research questions and interview guides

Themes in the protocol to be explored by the project
- *The process of diagnosis*
- Why a specific antibiotic was selected
- Where and how antibiotic were purchased
- Use of antibiotic,
- Satisfaction with antibiotic process
- Antibiotic knowledge
- Antibiotic attitudes
Example - Research questions pertaining to the theme *‘The process of diagnosis’*
- What symptoms made the patient seek the physician or the pharmacist?
- What did the patient want from the consultation?
- What did the patient expect from the consultation?
- Was the patient examined? If yes - how?
Example - Operationalized research questions in the interview-guide pertaining to ‘*The process of diagnosis’* (‘Patients with AB prescription’- please see appendix 1 for the full interview guide):
- When was the last time you got a prescription for an upper respiratory tract infection (should be within the last 3 months)
- What was the situation – what were your symptoms – for how long?
- Did you have any idea what kind of disease you were suffering from?
- Did you come here by your own initiative or were you encouraged by family, colleagues or friends?
- Which doctor did you seek – why this doctor?
- What did you want from the doctor?
- What did you expect from the doctor?
- How did the consultation go? What happened? What was said – by whom?
- Did the doctor make a diagnosis/examine you? If yes, how and do you know what the diagnosis was?

Finally, the protocol included practical guidance on how to recruit participants, how to conduct and transcribe the interviews and how to perform the first steps of the analysis (content analysis). Regarding recruitment, the protocol defined 4 groups that had to be interviewed, i.e., adults who were either patients or health care professionals, and delineated the research area into four specific ABs used for upper respiratory tract infections (URTIs). The use of the four selected ABs had been found to be particularly concerning in the quantitative consumption study [5], which is why they were considered interesting to investigate in depth; additionally, limiting the number of included ABs would increase the comparability of the data within the same country and between countries with regard to knowledge, attitudes and practices. The patients interviewed should have used one of the selected ABs for a URTI within 3 months, whereas the health care professionals (community pharmacists and general practitioners) were asked to recall 3 episodes in the last week in which they had managed some of the specified ABs for a URTI. The local data facilitators were encouraged to recruit participants with variations in gender, age and education and were further asked by the SSC to consider if they were in need of further inclusion criteria (for example: specific geographical areas, ethnic groups, language, etc.). Different sampling procedures were suggested, one of which was the snowball technique in which one’s professional and/or private networks are used to identify people who fulfil the inclusion criteria. It was emphasized that if this sampling procedure was used, it was not recommended for interviewers to interview people they knew personally. It was recommended in the protocol to make an appointment with patients to interview them some days after receiving their AB to investigate how they took the medicine. The protocol highlighted the ethical criteria involved in each step.

The protocol was written in English. As the interviews were conducted in the national language, this required both the translation of the developed interview guides into the local language and the translation of the transcripts of the conducted interviews back into English.

### The pilot project

Two-day pilot training seminars were carried out in November 2014 and January 2015 to ensure that the protocol initially developed by the SSC covered key aspects of the AB use culture as perceived by the pilot countries’ facilitators. Furthermore, the seminars were designed to train country facilitators to conduct the interviews. This was achieved by training them in the basics of qualitative research and ensuring that the SSC and the pilot countries had an aligned understanding of the content of the research questions in the protocol and interview guides.

Following the training sessions, each pilot country was instructed to conduct, transcribe and translate 3 interviews within each of the four groups of interviewees (patients who had recently had an AB prescription, patients who had recently bought an AB without a prescription, physicians and community pharmacists) according to the protocol.

Approximately 4 months after collecting the data, the pilot countries met with the SSC to complete the content analysis of the collected data.

### Evaluation

The pilot countries provided written and oral feedback on their experiences using Skype meetings, e-mail communication and face-to-face discussions. The feedback was focused on local ownership, research quality and feasibility:

Local ownership: Assessing if and how the participating countries experienced ownership of the project. As the SSC had taken the lead to form the project, it had to be ensured that this initiative did not undermine the commitment of the pilot facilitators.

Research quality: Assessing interview transcripts according to the principles of qualitative research, especially the skill to obtain narratives that provided coherent and detailed stories of interviewees’ experiences with AB [16]. For example, a full description of how patients purchased an AB in a pharmacy without a prescription would inevitably contain aspects of the patients’ knowledge and attitudes towards AB as well as both their and the pharmacists’ typical behaviour and social interactions.

Feasibility: Assessing challenges experienced with regard to the recruitment process, carrying out the interviews, documentation and analysis as well as an evaluation of the workload of facilitators.

The assessment was completed by the SSC and was based on the analytical principle of “critical common sense”, which Kvale describes as “interpretations that can be broader than the understanding of the interviewee him/herself, i.e., critically assess what is said” [17].

**Fig. 1:**
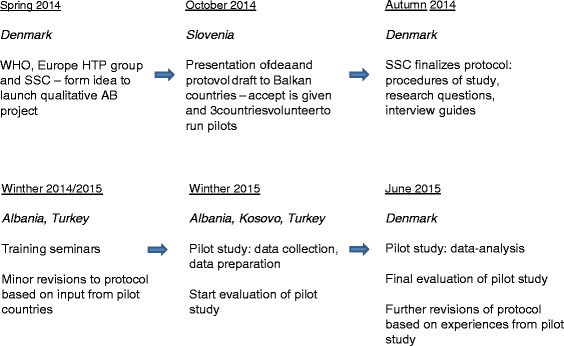
Overall depiction of the different steps of pilot study

Based on the evaluation, the SSC and pilot countries discussed different solutions to the identified challenges. Consensus was reached, and improvements were incorporated into the protocol (please see Fig. 1 under the heading October 2014 - Slovenia - there are some gabs between workds missing and an 'i' before 'idea' is missing for a full overview of the different steps of the pilot study).

### Ethics

No ethical approval was needed for the pilot evaluation as it consisted of the opinions and experiences of the authors of this article. However, the Danish Data Protection Agency has been approached in relation to the management and later publication of the data collected from the pilot projects.

## Results and discussion

Local facilitators in the pilot study achieved a sense of joint ownership. The protocol, including the interview guides, worked well. Several minor challenges pertaining to the research quality and feasibility were identified. These challenges were either a) challenges of qualitative research in general or b) challenges specific to conducting qualitative research in multi-country settings. In the following section, the challenges and solutions are presented and discussed in relation to methodological reflections on conducting qualitative research.

### Local ownership

The facilitators expressed feelings of joint ownership over the project. They stated that the interview guides and the project made sense to them partly as a result of having had the opportunity to provide feedback on the protocol initially developed by the SSC. They expressed a feeling of ownership over the data they provided, but not over the research process. They saw the project as a collaboration between three parties: the WHO, the SSC and their own country, and each party had a specific role to fulfil. The pilot countries were comfortable with this division of tasks.

The facilitators also felt that conducting the interviews had mostly confirmed their preconceived perceptions of the culture surrounding patients’ and health care professionals’ AB use. However, in some cases, the data collection process led to new insights; for example, some facilitators were surprised to learn that many patients were aware that ABs should only be used in some occasions. Finally, the facilitators stated that working in an open multi-country collaboration setting was helpful as they could discuss and learn from each other.

### Research quality

Challenges to research quality were identified. A general problem with interview flow was identified. Moreover, one specific challenge that arose was that the project facilitators in the pilot countries did not always manage to obtain narratives. Collaboration between the pilot countries and the SSC was found to be fruitful when analysing the data.

#### General challenges – flow of interviews

Facilitators felt that they had to repeat several questions, especially when asking health care professionals to describe three episodes from the last week in which they had managed AB use. According to the facilitators, this hindered the overall flow of the conversation.

#### Solutions – flow of interviews

In semi-structured interviews, it is of utmost importance for the interviewee “to talk freely and spontaneously about their feelings, experiences, attitudes and behaviour” [18]. As a result of this expressed challenge, changes to the interview guide were made. First, the number of recollections of specific AB episodes required by health care professionals was reduced from 3 to 2 episodes. Additionally, the facilitators suggested changing the order of questions for health care professionals.

#### Specific challenges – obtaining narratives

Obtaining narratives was feasible. One example was the story told by a father about his son, who had become ill. The father took the son to a general practitioner (GP), who concluded that the son was not ill enough to receive ABs. The GP orally instructed the father that if the situation deteriorated, the father should go to the community pharmacy and pick up a specific AB. The father was not given a prescription and was not instructed on how to use the AB should it become necessary to purchase it later on.

This example underlines the strength of recording narratives, as this short story provides insight into typical AB behaviours (GP promoting the use of AB without a prescription and some GPs appearing to provide only oral instructions and no information on the dosing regimen). The example also reveals underlying attitudes regarding AB use (GP thinks that not all situations should be treated with AB).

The transcripts also revealed that narratives were not always obtained. The transcripts were different in terms of the level of detail collected partly because not all local researchers had been authorized to record the interviews. However, even when recorded, narratives were not always provided. This could be due to the study design, which employed local facilitators who primarily had backgrounds in natural sciences.

#### Solutions – obtaining narratives

It was decided that from a workload perspective, no further interview training was feasible. Instead, the original protocol of 3 interviews within the 4 groups of interviewees (designed to balance data quality with feasibility) was changed to require 4 interviews per group. In this manner, more information about the AB culture would be revealed, not by conducting deeper interviews but by conducting more interviews. This compromised the workload of the data collectors but was necessary to ensure saturated themes regarding AB use.

Despite raising the number of interviews from 12 to 16, it was further deemed necessary to ensure the consistency of data and the possibility of achieving data saturation by having the interviewees address the same types of AB situations. The interview guides for the health care professionals were therefore revised, and they were now asked to recall 2 *typical* examples of AB consultations (and not just 2 consultations) during the last week.

#### Fruitful settings for analysing data

Coming from a different cultural context, the researchers from the SSC could guide the process of analysis as well as question the nuances of and reasons behind the AB knowledge, attitudes and behaviours revealed in the data. The pilot facilitators could correct and expand upon the SSC researchers’ understanding of the transcripts. Altogether, the collaboration provided more results, as well as more precise results, than if the parties had performed the analyses separately.

### Feasibility

The materials developed for the project worked well, and only minor adjustments had to be made. The major practical obstacle for the pilot facilitators was difficulty in recruiting patients. The local facilitators emphasized that it was a challenge for them to devote as much time to the project as they would have liked.

#### General challenges – recruitment of patients

Pilot facilitators used the snowball sampling technique to recruit patients but found it difficult to recruit enough patients. Furthermore, the facilitators discovered when conducting the interviews that patients sometimes had other types of infections that required ABs; however, due to a lack of physiological knowledge, they had agreed to be interviewed. There were no instances of incorrect recruitment due to patients having used a different AB than requested.

#### Solutions– recruitment of patients

The recruitment of participants is essential to the success of research projects [18], and difficulties in recruitment are common. Supplementing the snowball recruitment technique by offering financial incentives for participating patients was suggested. Using contacts at local community pharmacies was suggested as another method. These suggestions were added to the protocol.

To maintain a realistic workload balance for the facilitators, the SSC decided to include a small number of interviewed patients who were being treated for types of infections other than URTI as well as a few cases when the child was the one who was ill, as it was assessed that these interviews could still provide useful answers to the research questions.

#### General/specific challenges – interview guide

A few questions from the pilot countries were raised: a) the specific importance of and difference between certain questions in the interview guide and b) the experience that some questions in the interview guide had negative connotations. For example, the facilitators felt that it was patronizing to ask the patients: “Can you explain what an AB does in your body?”

These questions showed that the training sessions did not ensure that all questions in the interview guides and the nature of conducting qualitative interviews were understood in the same way by the SSC and pilot facilitators. Difficulties employing multiple interviewers can be found in all qualitative projects [19]; however, in this scenario, the challenge was likely reinforced by the multi-country setting.

#### Solutions – interview guide

Despite the recognition that a full acquisition of understanding between researchers is hard to achieve [19], it was assessed that the specific issues raised could be resolved. Hence, it was now explained more thoroughly in the protocol that interviewing allows for flexibility, for example, that questions can be phrased differently as long as they facilitate answers that can be used to answer the research questions of the study.

The identified challenges underlined the importance of closer contact between the SSC and the individual countries to discuss challenges that local facilitators experienced while conducting the interviews.

#### Specific challenges – allocating time

The facilitators in the pilot studies found it challenging to allocate a sufficient amount of time to the project. The local data collectors required between 15 and 80 min for the interviews (with some variation between the three pilot countries), with interviews with patients often being shorter than those with health care professionals. This timeframe was in many cases found to be acceptable; however, in some cases, this timeframe was too short, as some participants, especially patients, appeared to be in a hurry. Transcribing and translating the transcriptions were perceived to be the most time consuming tasks. This led pilot facilitators to ask the SSC for tighter instead of looser deadlines. It was therefore concluded that tighter timelines should be implemented.

### Limitations

We found both general and specific challenges with regard to conducting qualitative research in a multi-country setting; however, of these challenges, we identified only a few that were caused by the specific topic – culture of AB use. One such specific challenge that the pilot facilitators experienced was that patients agreed to be interviewed even when they suffered from the “wrong” infection or when it was the child and not the adult who was ill (despite these requests being stated in the protocol). In this study, most of these interviews were considered to contain valuable insight into the research questions and were therefore included in the analysis; however, this decision despite original intentions then to a small extent increased the heterogeneity of some of the data. As topic-dependent challenges were limited, we trust that most of the results of our evaluation are transferable to other qualitative multi-country research settings.

One study limitation that is probably transferrable to other settings was that several of the interviewers worked at national medical agencies, and it was thus expected that this would influence the answers of the interviewees, for example by omitting admissions of illegal conduct. This might certainly have influenced some results; however, surprisingly, most community pharmacists admitted that they sold ABs without prescriptions even though that is required by law. Hence, a high degree of truthfulness in many interviews was obtained.

The data collectors from Albania in particular had difficulties obtaining permission to record their interviews, which of course reduced the quality of the collected data, as using data recordings should be emphasized in all qualitative projects as the prime data collecting technique.

The validity of the evaluation of the study is believed to be high, as the results were discussed between the SSC and the pilot facilitators, and all contributed actively and equally to this publication.

## Conclusions

The aim of this paper was to contribute to the knowledge regarding how to conduct multi-country qualitative research. We propose that supporting previously untrained local facilitators to collect and analyse data is a means of achieving valuable research results. We recommend conducting formal discussions between research managers and local data facilitators to identify inadequacies in project design. Second, research managers should be prepared to adjust their protocols, both with respect to the depth and the breadth of the research, and to balance these aspects of research quality with the acquired skills of the enrolled researchers and their available time. Through testing, an acceptable balance can be achieved. Third, managers and data collectors should analyse the data together as this can contribute to highly valid results. Based on our experiences, we believe that qualitative multi-country research collaboration is possible when these issues are addressed. The results of this type of work could be of considerable importance for the health of populations worldwide.

## Appendix 1: Interview-guide – Patients with prescription

### Introduction

- Introduce yourself and introduce the study. Tell what the interview will be used for.
- Ask permission to record the interview on tape
- Assure anonymity of the interviewee
- Ask the interviewee to introduce her/him-self, including age, occupation and where they live

### Last face-to-face consultation with physician leading to prescription for an AB for an upper respiratory tract infection

#### Process of diagnosis of AB

- When was the last time you got a prescription for an upper respiratory tract infection (should be within the last 3 months)
- What was the situation – what were your symptoms – for how long?
- Did you have any idea what kind of disease you were suffering from?
- Did you come here by your own initiative or were you encouraged by family, colleagues or friends?
- Which doctor did you seek – why this doctor?
- What did you want from the doctor?
- What did you expect from the doctor?
- How did the consultation go? What happened? What was said – by whom?
- Did the doctor make a diagnosis/examine you? If yes, how and do you know what the diagnosis was?

#### Why a specific AB was chosen/Satisfaction with AB prescription process

- Who chose to use an AB?/Were you involved in this decision?
- If the doctor solely made the decision – was any explanation given why to use an AB?
- What AB was chosen? (with regard to product name, form and strength)
- Were you involved in the decision of what specific AB to use?
- If yes, please describe how. If the doctor chose the AB, did he/she explain why to use this specific AB?
- Did the doctor give any instructions on how to use the AB?
- If yes – which ones? Were the instructions given written or orally?
- Were you satisfied with the way the consultation went?
- If yes, why? If no, why not?

#### Where was the AB purchased/Satisfaction with AB purchase process

- Where did you purchase the AB prescribed by your GP?
- Why did you choose this place?
- Please describe the circumstances of the purchase? What happened? What was said – by whom?
- Were you involved in which specific AB was purchased? (due to generic substitution or availability of the drug in the etc.) If yes, please describe how.
- Did the pharmacist give any instructions on how to use the AB? If yes – what were they?
- Were the instruction given written or orally?
- Were these instructions similar to the ones provided by the doctor?
- If the pharmacist changed your medicine to another brand - how did you feel about this?
- Were there any challenges with regard to the purchase in terms of price; was the drug in stock in the store, etc.?
- How much medicine did you purchase? Did you purchase all the necessary AB immediately? If not, why so?
- Were you satisfied with the way the purchase went? If yes, why? If no, why not?

#### Use of AB/Satisfaction with AB use

- When you came home - how did you use the AB? (How many tablets/dosages per day for how long?)
- Did this use correspond with the instructions given by the doctor or pharmacist? If yes, why? If no, why not?
- Did you experience any challenges with taking the AB? If yes, which ones?
- Did the AB alleviate or cure your symptoms? If yes, please explain how and how quickly?
- Did the original symptoms reoccur?

### AB use in general

#### The last time compared to other times

- Have you had AB prescribed before?
- If yes, approximately how many times? When did it take place?
- What were the symptoms – for how long?
- Did you have any idea what kind of disease you were suffering from?
- Did the consultations you had with your doctor at these times resemble the last time you received a prescription? Did you also receive a prescription at these times?
- Was the diagnosis carried out the same way as the last time you had an AB consultation? If no, describe how it usually took place.
- Was the way the AB was chosen previously similar to the last time you had an AB prescription? If no, please describe how it usually took place.
- Did the doctor give similar instructions about how to use AB compared to the last time you had an AB prescription? If no, please describe how it usually took place.
- Did you suffer previously from any of these symptoms without seeking a GP? If yes, why (at these times) not think it was necessary to seek a GP?
- Did the visits to the pharmacy where you previously purchased your AB prescription resemble the last time you purchased the AB?
- Were you previously involved in which AB was purchased in the same way as the last time you purchased an AB If no, please describe how it usually took place.
- Did the pharmacists previously give you instructions about AB use in the same way as the last time you purchased an AB? If no, please describe how it usually took place.
- The way you took your AB treatment – does this resemble how you took AB before (number of days, compliance with advice of GP or pharmacist, etc.)

#### Knowledge and attitudes about when to use AB

- Can you explain what AB does in your body?
- From where do you have this knowledge?
- In which situations do you think AB should be used?
- Why do you think that AB should be given in these situations?
- Where do you have this knowledge from? Do you ever discuss these issues with family, friends, colleagues? If so, do you all agree on these matters?
- Are there situations in which you think AB should not be used?
- Which situations are these?
- Why do you think that AB should not be used in these situations? Where do you have this knowledge from?/Do you ever discuss these issues with family, friends, colleagues? If so, do you all agree on these matters?
- Have you ever seen public campaigns addressing AB use? If yes, what can you remember from these campaigns? Did the campaigns affect you in any way? If yes, in which way? If no, why not?

### Finalizing interview

- Thank the interviewee for their time.
- Ask if they have any additional comments to what was said during the interview
- Tell the interviewee what will happen to the recordings now
